# Engineering Multilayer Poly(ethylene glycol) Hydrogels via Off-Stoichiometry Thiol–Ene Chemistry

**DOI:** 10.1021/acsapm.6c01912

**Published:** 2026-07-20

**Authors:** Ridhi Pradhan, Ravinun Khamleart, Waqas Saleem, Michael J. McShane

**Affiliations:** † Department of Biomedical Engineering, 14736 Texas A & M University, College Station, Texas 77843, United States; ‡ Department of Biomedical Engineering, 26685 Mahidol University, Nakhon Pathom 73170, Thailand; § Department of Materials Science and Engineering, Texas A & M University, College Station, Texas 77843, United States; ∥ Center for Remote Health Technologies Systems, Texas A & M University, College Station, Texas 77843, United States

**Keywords:** off-stoichiometry thiol–ene, multilayer hydrogel, Michael addition, interfacial adhesion, compartmentalization, biomaterials, spatially controlled mechanics

## Abstract

Multilayer hydrogels with spatially controllable mechanical and functional properties are increasingly valuable, yet their utility is often constrained by fabrication challenges such as weak interfacial adhesion as well as reliance on cytotoxic photoinitiators and UV exposure. This study presents a simple, robust, and photoinitiator-free method to engineer multilayer poly­(ethylene glycol) (PEG) hydrogels using off-stoichiometry thiol–ene (OSTE) Michael addition chemistry. By deliberately utilizing an alternating thiol-rich and vinyl sulfone-rich PEG formulation, residual functional groups are preserved at the layer interfaces and subsequently form covalent bonds between the adjacent layers, enabling strong interfacial adhesion without UV irradiation, photoinitiators, or adhesives. The resulting hydrogels exhibited rapid and tunable gelation (1–5 min), with mechanical properties spanning ∼0.5–6 kPa through varying polymer concentration (5–10 wt %) and thiol–ene ratio (0.6–1.6). Ellman’s assay confirmed the presence of stoichiometry-dependent unreacted thiol groups, validating the proposed interfacial bonding mechanism. Peel tests revealed strong interfacial adhesion, with failure occurring within the bulk material rather than at the interface. Nanoindentation further revealed spatially defined stiffness profiles across the multilayer construct, confirming precise mechanical compartmentalization. The hydrogels displayed tunable swelling behavior and degradation profiles depending on the formulations. Additionally, all single- and multilayer hydrogels maintained high cytocompatibility (>90% NIH/3T3 mice fibroblast viability) independent of polymer concentration, thiol–ene ratios, or layering. Incorporation of alginate microparticles provided an additional strategy for modulating hydrogel properties, significantly reducing swelling and enhancing stiffness while preserving structural integrity. Overall, the findings established a platform for engineering heterogeneous hydrogel constructs with spatially controlled properties, biofunctionality, and compatibility features that make them relevant for biomedical applications.

## Graphical abstract

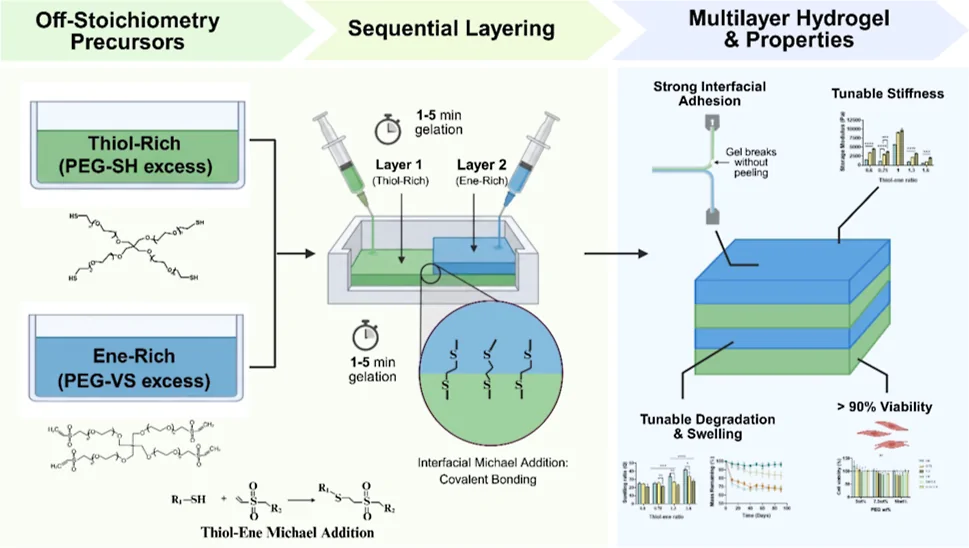

## Introduction

Hydrogels are three-dimensional, cross-linked polymer networks capable of absorbing and retaining large amounts of water while preserving their structural integrity. Their soft, hydrated, and viscoelastic properties closely mimic those of native biological tissues, which has led to their investigation for applications including drug delivery, tissue engineering, biosensing, and wound healing.^1^ However, conventional hydrogels typically feature homogeneous architectures, which limit their performance in complex physiological environments. In many settings, spatial variation in parameters such as mechanical stiffness, degradation rate, and biomolecular release is critical for replicating tissue heterogeneity and achieving precise therapeutic outcomes.^2^

To address these limitations, multilayer hydrogel systems have been developed, offering compartmentalized and programmable functions through discrete layers with tunable properties. Such architecture enables spatial control over mechanics and biochemical presentation, opening opportunities for gradient-driven cell culture, controlled drug release, and multiplexed sensing.^2–7^ Despite these advances, fabrication of multilayer hydrogels remains challenging, particularly when layers with different compositions must be joined while maintaining interfacial stability. Insufficient interfacial bonding can lead to delamination, reduced mechanical integrity, and altered transport or release characteristics.^2,6^

Poly­(ethylene glycol) (PEG)-based hydrogels have been frequently used because of their biocompatibility, hydrophilicity, and ease of chemical modification. Photopolymerization remains one of the most widely used methods for fabricating multilayer PEG hydrogels because it enables rapid network formation and spatial control over cross-linking. In many multilayer PEG systems, individual layers are sequentially polymerized using ultraviolet (UV) light and photoinitiators to allow attachment of subsequent layers. Residual photoinitiators and their photolysis products also require removal following fabrication, as their presence can influence the cytocompatibility of the resulting hydrogel.^8^ Although this approach has enabled the fabrication of gradient and multilayer constructs, it requires exposure to UV light and photoinitiators, and fabrication outcomes can depend on precise control of factors such as light penetration depth, exposure time, and construct thickness.^9,10^

Alternative cross-linking approaches have therefore been investigated for hydrogel fabrication. Among these, thiol–ene “click” reactions, particularly thiol Michael addition to vinyl sulfone, can proceed under mild aqueous conditions without the use of photoinitiators. These reactions enable tunable gelation kinetics and uniform cross-linking, and these systems have been explored for applications involving cell encapsulation and other biomedical uses.^11–15^

In this work, we investigated the fabrication of multilayer PEG hydrogels using an off-stoichiometry thiol–ene (OSTE) system comprising four-arm PEG vinyl sulfone (PEG-VS) and four-arm PEG thiol (PEG-SH). By deliberately employing stoichiometric imbalance, thiol-rich or vinyl sulfone-rich compositions in alternating layers, unreacted functional groups are preserved at the interfaces. These groups can participate in subsequent interfacial reactions during layer addition, providing a mechanism for covalent bonding between adjacent hydrogel layers without the use of photoinitiators, UV irradiation, or external adhesives.

In this work, we explored the feasibility of this fabrication strategy and conducted comprehensive characterization of multilayer constructs prepared with varying thiol–ene ratios: mechanical and rheological tests quantified stiffness and tunability across stoichiometries; adhesion testing was used to assess interlayer bonding strength; assays confirmed unreacted functional groups for robust interlayer bonding; swelling and degradation studies evaluated stability under physiological conditions; and cytocompatibility was assessed using NIH/3T3 fibroblasts to validate suitability for biomedical applications. Together, the results provide insight into the influence of thiol–ene stoichiometry on multilayer hydrogel formation, interfacial bonding, and material properties relevant to biomedical applications.

## Experimental Section

### Materials

Four-arm PEG vinyl sulfone (10KDa, PEG-VS) was purchased from Jenkem Technology. Four-arm PEG-thiol (10KDa, PEG-SH) was purchased from Laysan Bio, Inc. Phosphate-buffered saline (PBS) tablets (lot no. 19A1456362) were purchased from VWR Chemicals, LLC, OH. 5,5′-Dithiobis­(2-nitrobenzoic acid) (DTNB, Ellman’s reagent) (Cat: 117540010) and triethanolamine (TEA, Cas102-71-6) were purchased from Thermo Scientific. Ethylenediaminetetraacetic acid (EDTA) and cell counting kit-8 (CCK-8) were purchased from Millipore Sigma.

### Hydrogel Synthesis: Preparation of a Single-Layer Hydrogel

The PEG hydrogels were synthesized by cross-linking four-arm PEG-SH (10 kDa) and four-arm PEG-VS (10 kDa) via the Michael addition thiol–ene reaction. Precursor solutions were prepared at varying polymer concentrations of 5%, 7.5%, and 10 wt % and thiol-to-ene molar ratios of 0.75:1.25 (0.6), 0.75:1 (0.75), 1:0.75 (1.3), and 1:1.25 (1.6). Cross-linking was initiated by adjusting the pH of the mixture to 8.0–8.5 using 50 mM triethanolamine (TEA). For gel casting, 400 μL of precursor solution was pipetted between glass slides separated by Teflon spacers (0.75 or 1.5 mm) and allowed to polymerize at room temperature. Following gelation, the hydrogels were carefully removed, equilibrated in PBS for at least 24 h, and stored at 4 °C until further testing.

### Hydrogel Synthesis: Preparation of Multilayered Hydrogels

Multilayer PEG thiol–ene hydrogels were fabricated using a sequential layer-stacking approach. Each construct was composed of alternating layers formulated with either excess thiol or excess vinyl sulfone groups. For the first layer, PEG-SH and PEG-VS were mixed at ratios of 1.25:0.75 (1.6) or 1:0.75 (1.3) to a final volume of 400 μL. The pH of the precursor solution was adjusted to 8.0–8.5 using 50 mM TEA, and the mixture was cast between glass slides separated by 0.75 mm Teflon spacers. After polymerization at room temperature, the top glass slide was carefully removed, and an additional 0.75 mm spacer was stacked onto the existing spacer. The second prepolymer solution (PEG-SH:PEG-VS at 0.75:1.25 (0.6) or 0.75:1.0 (0.75), 400 μL) was then added above the polymerized layer and allowed to cure. This procedure was repeated iteratively to generate multilayer hydrogels with the desired number of layers [Figure 1].

**Figure 1:**
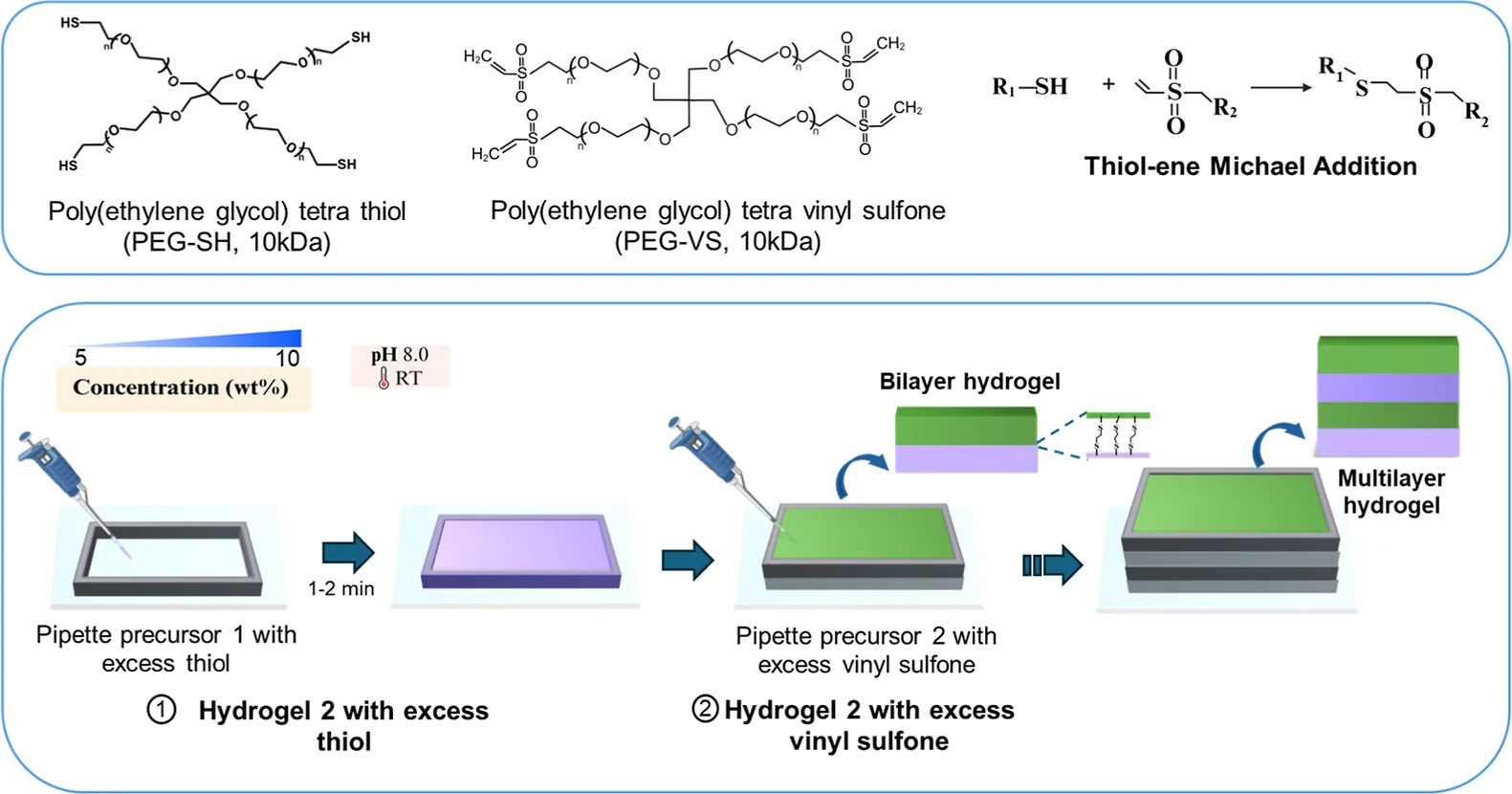
Schematic of the sequential fabrication of multilayer PEG hydrogels using thiol–ene click chemistry. Steps (1) and (2) are repeated to achieve multilayer hydrogels with strong bonding between adjacent layers presenting excess thiol and sulfone moieties.

### Swelling Studies

The swelling experiments were conducted on preswollen samples (*n* = 4) that had been equilibrated in PBS at pH 7.2 for 48 h. After weighing the swollen samples, they were desiccated in a vacuum desiccator for 48 h, and then their dry weights were recorded. The equilibrium swelling ratio was calculated using

$$Swelling\;ratio=\frac{W_{s}-W_{D}}{W_{D}}$$

where *W*_S_ and *W*_D_ represent the weight of the hydrogel in the swollen state and dry state, respectively.

### Rheology and Gelation Kinetics

Hydrogels with varying PEG wt % and thiol–ene ratios were swollen in PBS at pH 7.2 for 48 h. Subsequently, 8 mm disks were punched out from the swollen hydrogels. Frequency and amplitude sweeps were performed using a PP08 parallel plate Anton Paar Rheometer to identify the linear viscoelastic region, with a plate diameter of 8 mm, and a gap was set to 1.5 mm. Following this, a time sweep was conducted under 1% strain and 1 rad s^–1^ angular frequency at 37 °C. The storage modulus of each sample (*n* = 4) was calculated as the average storage modulus over 60 s during the time sweep at 1% strain and 1 rad s^–1^.

The gelation kinetics of these hydrogels were investigated using a Rheometer Anton Paar. 60 μL of prepolymer solution (*n* = 4) was pipetted onto an 8 mm parallel plate tool. Subsequently, a time sweep was performed at room temperature under 1% strain and 1 rad s^–1^. The gel point was determined as the crossover point between storage modulus (*G*′) and loss modulus (*G*”), after which *G*′ sharply exceeded *G*”.^16^ Time zero was defined as the initial of rheological data acquisition immediately following the mixture of the precursor components and loading onto the rheometer. The rheometer gap was fixed at 800 μm.

### Degradation Study

5 mm of swollen PEG SH-VS hydrogels with different formulations were transferred to 2 mL tubes (*n* = 4). Each tube was filled with 1 mL of PBS (pH 7.4) and incubated at 37 °C for hydrolytic degradation studies. The weight of each sample (*W*_i_) was taken and remeasured (*W*_r_) every 10 days for 90 days. The PBS medium was replaced after each measurement. Similarly, the hydrogels were incubated at 37 °C in 1 mL of 3% hydrogen peroxide (H_2_O_2_) with 1.28 mM of cobalt chloride (CoCl_2_) for accelerated oxidative degradation. Under these conditions, the weight measurements were taken every 30 min until complete hydrogel degradation.

The hydrogels were suspended in fresh buffer following each measurement. The remaining weight percentage of the samples was calculated using

$$wt.\;remaining\;\left(\%\right)=\frac{W_{r}}{W_{i}}\times 100\%$$

where *W*_i_ is the mass of the hydrogels that did not undergo degradation and *W*_r_ is the mass of the hydrogel at time points.

### Determination of Unreacted Thiol Groups

To determine the unreacted thiol remaining in the hydrogels, the amount of free thiol was quantified with Ellman’s reagent (DTNB). The DTNB reacts with thiol groups, forming a yellow dye known as 5-thio-2-nitrobenzoic acid (NTB) that can be quantified spectroscopically at 412 nm.^17,18^ 25 μL of hydrogels (*n* = 3) with different formulations were prepared and swelled in Ellman’s reaction buffer (270 μL) containing 0.1 M sodium phosphate and 1 mM ethylenediaminetetraacetic acid (EDTA) at pH 7.5–8. Then, Ellman’s reagent (5.4 μL, 4 mg in 1 mL reaction buffer) was added to wells containing the gels and placed in an orbital shaker in the dark for 90 min or until the gel color matched the supernatant, ensuring diffusion of the yellow NTB^2–^ dianion out of the gel. The absorbance for each condition was measured at 412 nm.

### Characterization of Adhered Hydrogels

#### T-Peel Test of Layered Hydrogels

The peel strength between hydrogels with different polymer compositions was determined by performing T-peeling tests with a mechanical testing machine (DMA Q800). As part of the sample preparation process (5, 7.5, and 10 wt %), after the fabrication of the first layer, 1/3 of the hydrogel was covered with parafilm to prevent interfacial adhesion between two gel layers. Then, the precursor for the second layer was stacked onto the first layer. The samples, each with a thickness of 1.5 mm per layer, were cut to specific dimensions (10 × 70 mm) and then incubated in PBS for 48 h. To fix the back of the gels, a tape was adhered to the outer side of the gels. The peel arm of the bilayer gel was fixed to the upper clamp (movable clamp), and the other arm of the gel was clamped to the lower clamp. The gels were then peeled at a peeling rate of 10 mm/min.

### Nanoindentation (Stiffness)

The Young’s modulus of the layered hydrogels was measured under uniaxial compression using an Optics 11 Life Pavone nano indenter equipped with a spherical probe (25 μm diameter, 0.025 N/m stiffness). Hydrogels were cast in rectangular Teflon molds (4 × 3 × 0.75 mm), with sequential layers prepared in thiol–ene ratios of 0.6, 1.3, 0.75, and 1.6 (Figure 3c).

To ensure stable adhesion during testing, hydrogels were mounted on functionalized glass coverslips. Coverslips were treated with (3-mercaptopropyl)­trimethoxysilane by immersion in a solution of 1% silane and 95% ethanol (pH 4.0–4.5) for 10 min, followed by drying at room temperature for 24 h.^19^ Then, 30 μL of the precursor thiol–ene hydrogel was pipetted onto the silanized coverslip, and the preformed hydrogel sample (*n* = 3) was gently mounted on top prior to complete cross-linking. The coverslip–hydrogel assembly was affixed to a 24-well plate for indentation. Mechanical testing was performed in PBS at 25 °C, with indentation depths set to 2 μm.

### Cytotoxicity

NIH/3T3 mouse embryonic fibroblast cells at passage 24 were cultured in DMEM supplemented with 10% FBS and 1X P/S antibiotics. Cells were maintained in a humidified incubator at 37 °C with 5% CO_2_. Cytotoxicity testing was carried out following the manufacturer’s protocol (CCK-8 test). Briefly, after achieving 80% confluency, cells were trypsinized and seeded at a density of 5000 cells per well in 96-well plates, with 100 μL of complete DMEM. After allowing cell adherence for over 24 h, one-beam sterilized PEG hydrogel strips per well (*n* = 3) (5 × 0.5 × 0.5 mm) with different polymeric concentration (5, 7.5, and 10 wt %), thiol–ene ratio (0.6, 0.75, 1.3, and 1.6), and layers (single or double, 0.6/1.6 represents first hydrogel with 0.6- and second layer with 1.6 thiol–ene ratio) was added to the well for the next 24 h and incubated at 37 °C with 5% CO_2_. Following the treatment, 10 μL volume of CCK-8 reagent was added to each well, and absorbance was measured at 450 nm after 3 h. Percentage viability for each sample was calculated via normalization to the mean of the blank control group.

### Statistical Analysis

Data are shown as the mean ± standard deviation. Statistical analyses were performed using GraphPad Prism. Normality was accessed using the Shapiro–Wilk test, and homogeneity of variance was evaluated using Bartlett’s test prior to parametric testing. One-way ANOVA followed by Tukey’s posthoc test was used when the assumptions were satisfied, while the Kruskal–Wallis test with Dunn’s posthoc correction was applied when assumption was not met (ns *p* > 0.05, *p ≤ 0.05, **p ≤ 0.01, ***p ≤ 0.001, ****p ≤ 0.0001).

## Results and Discussion

### Gelation Kinetics and Mechanical Properties

The *G*′ of PEG hydrogels began increasing immediately after mixing the precursor across all tested formulations, with gel points ranging from 1 to 5 min, depending on precursor concentration. Varying the thiol–ene ratio did not impact gel time, but polymer concentration did: the quickest gelation was observed at 10 wt % (1.1 ± 0.06 min) versus 5 wt % (3.75 ± 0.22 min) (Figure 2a). For all formulations, *G*′ exceeded *G*″, confirming network formation and primarily elastic behavior. The observed range of gelation times may be relevant for applications requiring control over the working time prior to gel formation. For instance, injectable hydrogels need slower kinetics to prevent needle clogging but should also gel rapidly enough to stay localized.^20^ Similarly, rapid gelation helps ensure that bioactive agents, such as cells, enzymes, or drugs, are evenly distributed within the gel matrix before complete cross-linking.^21^ The mechanical properties of the hydrogels were evaluated using a rheometer at a fixed frequency of 1 rad s^–1^ and a strain of 1%. These properties were found to be tunable by adjusting either the polymer concentration or the thiol–ene ratio. For hydrogels with a 0.6 thiol–ene ratio, equilibrium storage modulus values were 1479.3 ± 22, 3444.5 ± 79, and 4532.4 ± 108 Pa for 5, 7.5, and 10 wt % polymer concentrations, respectively. Hydrogels with a 0.75 thiol–ene ratio demonstrated equilibrium storage moduli of 1130.7 ± 40, 2963.8 ± 123, and 3694.5 ± 213 Pa for 5, 7.5, and 10 wt % concentrations, respectively. Similarly, hydrogels with a 1.3 thiol–ene ratio yielded moduli of 917.3 ± 56, 2166.1 ± 43, and 3266.1 ± 108 Pa, and for a 1.6 thiol–ene ratio, the values were 492.1 ± 64, 901.1 ± 15, and 2076.9 ± 145 Pa for 5, 7.5, and 10 wt % concentrations, respectively. Oscillatory shear measurements indicated that increasing PEG concentration in the precursor solution significantly enhanced the storage modulus of these hydrogels. These trends can be attributed to the increasing concentration of PEG-SH and PEG-VS which directly increases the concentration of the cross-linking moieties in the hydrogel network.^22–24^ Additionally, rheological characterization of these hydrogels revealed a maximum storage modulus at the stoichiometric 1:1 thiol-to-vinyl sulfone ratio, consistent with the expectation that balanced functional group concentrations maximize network connectivity in step-growth polymerizations. Interestingly, off-stoichiometric formulations exhibited a pronounced mechanical asymmetry. Hydrogels formulated with excess PEG-VS (0.75:1.25 and 0.75:1.0) retained substantially higher storage moduli than the corresponding PEG-SH excess formulations (1.25:0.75 and 1.0:0.75). This observation suggests that deviations from stoichiometric balance affect the network architecture differently depending on which functional group is present in excess.^25–27^

**Figure 2:**
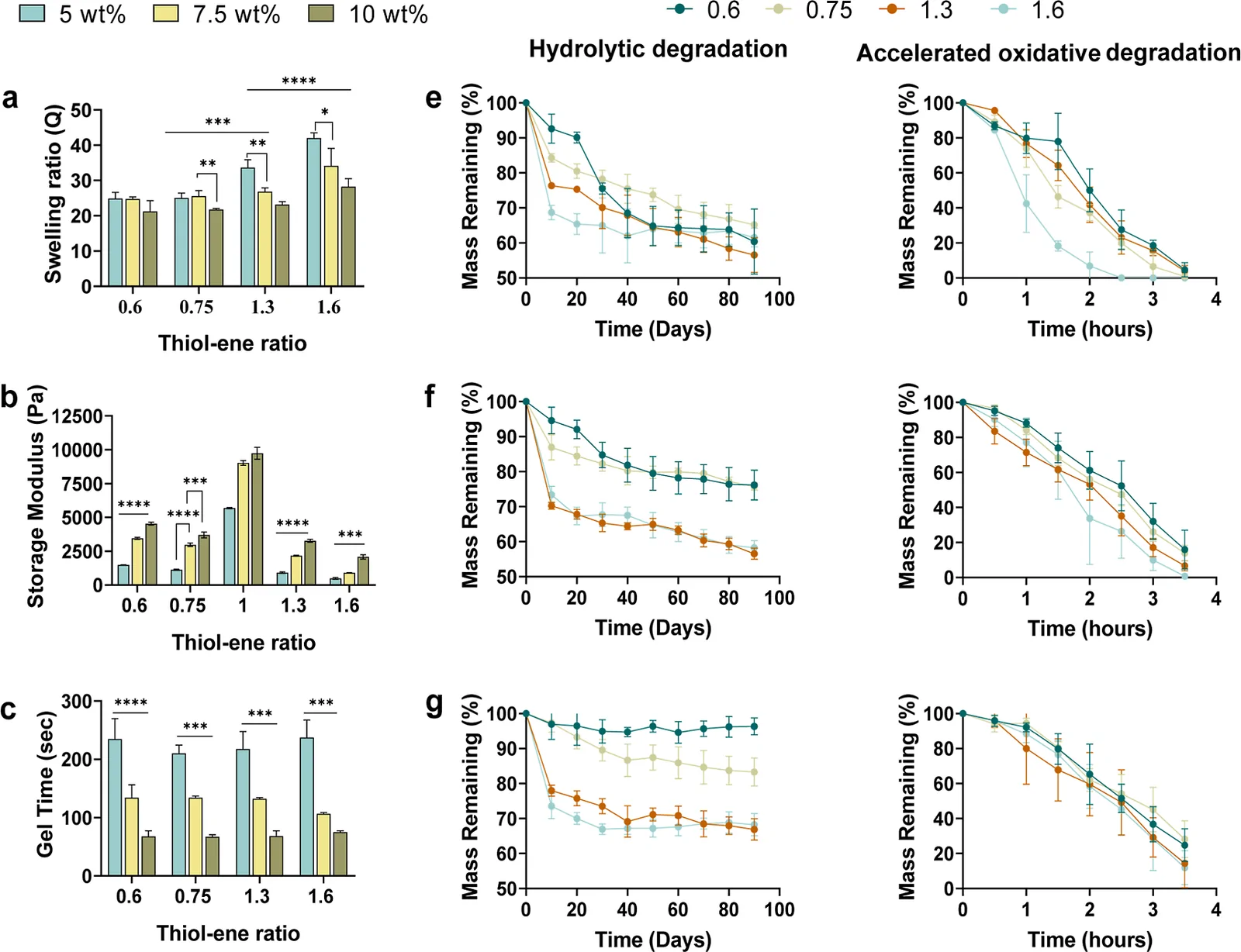
Characterization of PEG-based thiol–ene hydrogels with varying polymer concentrations and thiol–ene ratios. (a) Swelling ratio, (b) storage modulus (*G*′), and (c) gelation time of hydrogels at different thiol–ene ratios and polymer concentrations. (e–g) Degradation profiles of hydrogels under hydrolytic (PBS, 37 °C) and accelerated oxidative (3% H_2_O_2_ with 1.28 mM CoCl_2_) conditions and (e) 5 wt %, (f) 7.5 wt %, and (g) 10 wt % hydrogels. Error bars represent one standard deviation (*n* = 4). Statistical significance is indicated: *p < 0.05; **p < 0.01; ***p < 0.001; ****p < 0.0001.

In the PEG-VS excess formulations, unreacted vinyl sulfone groups do not significantly homopolymerize under these conditions, and they remain as unreactive pendant ends that do not reinforce the network. Instead, the higher modulus at 0.6 (0.75:1.25 thiol–ene) is driven by the kinetics of multiarm network formation and the minimization of primary loops.^17,28^ As gelation proceeds in 4-arm PEG systems, increasing connectivity restricts macromolecular mobility, making cross-linking severely diffusion-limited.^29^ The greater local concentration of vinyl sulfone increases the statistical probability of productive intermolecular interactions. This stoichiometric excess prevents propagating thiols from forming elastically inactive intramolecular loops or being sterically trapped. Consequently, the 0.75:1.25 formulation converts a higher fraction of its limiting thiols into active network junctions, yielding a higher effective cross-link density.

In contrast, excess thiol groups exist in equilibrium with their corresponding thiolate species under the mildly basic conditions employed for Michael addition. Thiolates are susceptible to oxidation and may participate in competing side reactions, including disulfide formation. Such reactions can potentially promote loop formation or other network defects that do not contribute to elastic load bearing. The more pronounced reduction in storage modulus observed for thiol-rich formulations is therefore consistent with a greater loss of effective network connectivity arising from both off-stoichiometric cross-linking and possible thiol-mediated side reactions.^30–32^

Again, the mechanical properties must be considered in the context of the intended application. For instance, softer hydrogels have shown enhanced cell survival and extracellular matrix elaboration due to the larger mesh size.^33^ Additionally, the lower stiffness of the PEG hydrogel is desirable as hydrogels with lower stiffness led to reduced macrophage activation and less severe and more typical FBR, making it suitable for in vivo biomedical applications.^33^ However, for implantation purposes, while being soft and flexible, the hydrogel should also maintain structural integrity and mechanical stability over time. It must maintain the desired shape, as well as withstand the mechanical stress present in the tissue environment.

### Equilibrium Swelling and Degradation Studies

Swelling and degradation behavior are key factors influencing the permeability and release of biomolecules in hydrogels. A higher swelling ratio leads to an increased mesh size, which can enhance the diffusion of nutrients, bioactive molecules, and waste through the hydrogel network. The equilibrium swelling ratios of hydrogels with different formulations are compared in Figure 2c. In general, the swelling ratio decreased significantly (*p* < 0.0001) as the PEG concentration increased from 5, 7.5, and 10 wt % for thiol–ene ratios of 1.3 and 1.6 (33.7 ± 2.2, 26.8 ± 1.1, and 23.2 ± 0.8 compared to 42.0 ± 1.4, 34.1 ± 4.9, and 28.2 ± 2.3). This trend can be attributed to the increased cross-link density present at higher polymer concentrations. In contrast, the swelling ratio was not significantly affected by variations in PEG concentration for 0.6 and 0.75 thiol–ene ratios (24.9 ± 1.7, 24.7 ± 0.6, and 21.1 ± 3.0; 25.0 ± 1.5, 25.5 ± 1.6, and 21.8 ± 0.3). This polymer concentration effect observed at higher thiol–ene ratios may be attributed to the relatively greater hydrophilicity of thiol compared to vinyl sulfone enhancing the water uptake.^34,35^

For hydrolytic degradation studies (Figure 2e–g), the gels were incubated in PBS at 37 °C. Gels made with 10 wt % polymer concentration with a higher thiol–ene ratio showed degradation up to 30%, in contrast to gels made of a lower thiol–ene ratio that were found to be much more stable (degradation of 5% in 90 days). This trend was consistent in hydrogels with 5 and 7.5 wt %. where a lower thiol–ene ratio yielded slower degradation. The 7.5 and 5 wt % hydrogels showed degradation up to 44% and 40%, respectively, in 90 days. Excess thiol groups may promote dangling chains, intramolecular loops, and other network defects that reduce effective cross-link density, increase mesh size, and accelerate hydrolytic degradation. In contrast, excess vinyl sulfone groups are expected to remain largely as pendant functionalities that minimally disrupt network connectivity. These observations are consistent with the rheological results, which also showed lower storage moduli for thiol-rich formulations, supporting the role of network architecture in governing hydrogel stability. Overall, the slower degradation of these in PBS can be due to the stability of ether linkages as hydrolysis cleaves ester bonds, which are absent in these polymers.^36^ Additionally, an increase in polymeric concentration resulting in increased cross-linking density has also shown to slow degradation in PEG hydrogels. On the other hand, increasing hydrophilicity and swelling also generally result in more rapid hydrolysis.^37,38^

As noted, the oxidative degradation of the PEG hydrogels was accelerated by using an aqueous solution of 3% hydrogen peroxide with 1.25 mM cobalt chloride. The cobalt ions rapidly decompose hydrogen peroxide to produce highly reactive hydroxyl (OH) radicals via the Haber–Weiss reaction; this has been shown to degrade polymers oxidatively.^39,40^ All of the hydrogel formulations experienced similar oxidative degradation profiles. This was expected as the ether backbone of PEG is susceptible to oxidative cleavage. The gels completely dissolved after 4 h, with 5 wt % gels exhibiting the fastest degradation rate. These were followed by 7.5 and 10 wt %, respectively, as shown in Figure 2e–g. It is relevant to note that, for in vivo applications, the polyether polymers are susceptible to oxidative degradation due to the ROS production by the adherent macrophages and foreign body giant cells. However, PEG has antifouling properties and biocompatibility, so there is less chance of oxidative degradation due to adherent cells after implantation.^41^ Browning et al. demonstrated that even though PEG-based hydrogels underwent measurable oxidation during in vitro degradation studies, degradation of PEG hydrogels in vivo is primarily driven by hydrolysis degradation when implanted in subcutaneous tissues.^42^ Additionally, in vivo degradation rates are also dependent on the implant location as well as the geometry of the specific implant.^43^

## Determination of Unreacted Thiol Groups

The unreacted thiol content of the hydrogels was quantified using Ellman’s reagent. As shown in Figure 3a, no absorbance was observed with the hydrogels formulated using 0.6 and 0.75 thiol–ene ratios. In contrast, the absorbance at 412 nm was pronounced for thiol–ene ratios 1.3 and 1.6 and increased with polymer concentration from 0.16 to 0.3 for 5 wt %, 0.19 to 0.32 for 7.5 wt %, and 0.25 to 0.39 for 10 wt %. There was a significant difference between the absorbance of the hydrogel with 1.3 and 1.6 thiol–ene ratios, as expected.^18^ The 1.6 thiol–ene ratio has more unreacted thiol groups; thus, the absorbance was found to be higher by >50% in all polymeric concentrations compared to 1.3 thiol–ene. Although the 10 wt % formulation contains a higher theoretical excess of thiol groups than the 5 wt % formulation, Ellman’s assay quantifies only accessible residual thiols rather than total theoretical thiol content. Therefore, the measured signal may not scale linearly with stoichiometric excess due to diffusion limitations within the hydrogel network, local microenvironment effects, and incomplete accessibility of all thiol groups. This finding supported the concept of bonding unreacted thiol groups to unreacted alkene groups for fabricating multilayered hydrogels with adherent interfaces.

**Figure 3:**
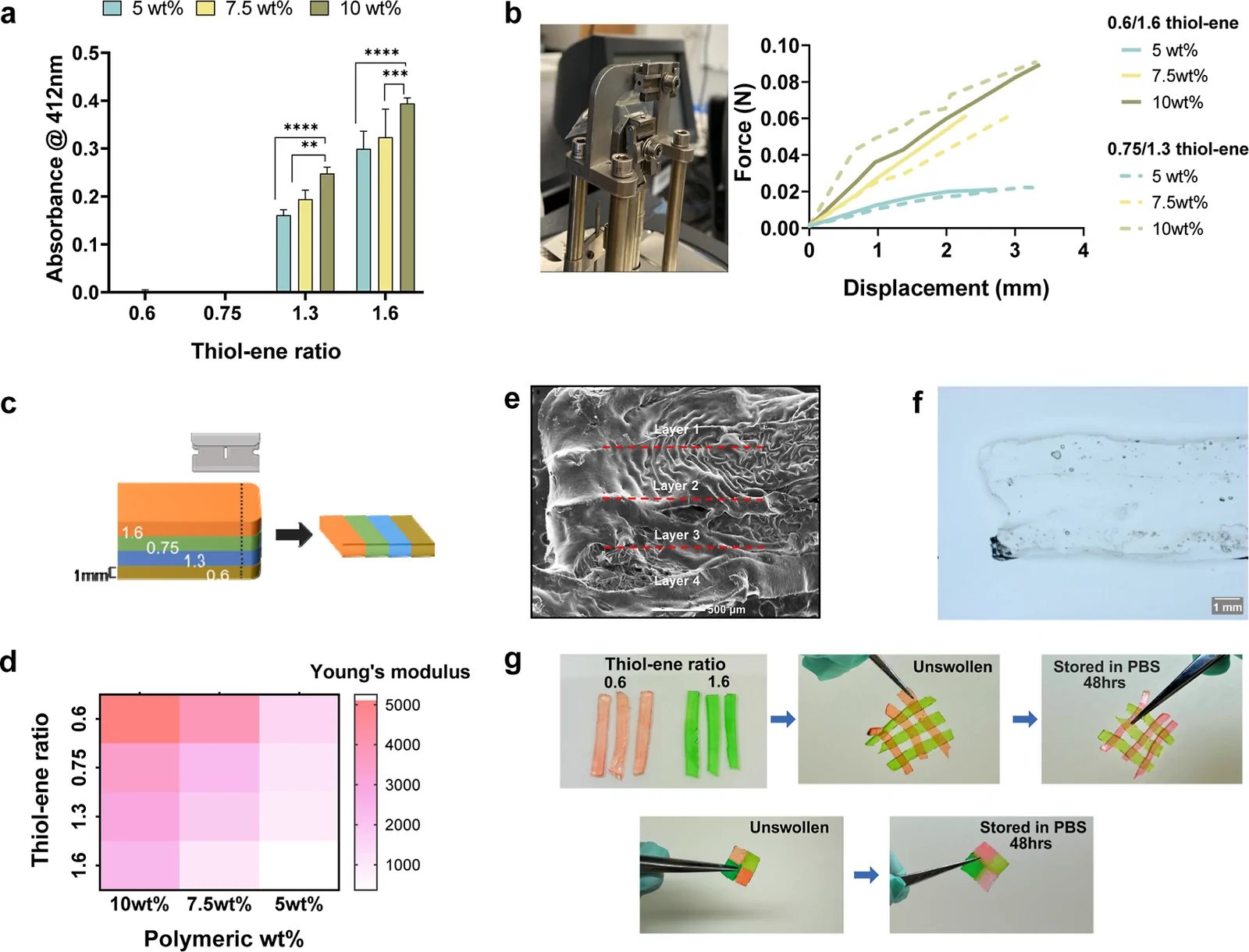
Evaluation of interfacial bonding and mechanical characterization of multilayer PEG hydrogels. (a) Quantification of unreacted thiol groups using Ellman’s reagent (*n* = 3). (b) T-peel test setup and representative force–displacement curves for bilayer hydrogels composed of alternating thiol-rich (1.6) and ene-rich (0.6) layers, or 1.3 and 0.75 thiol–ene ratios, prepared at polymer concentrations of 5, 7.5, and 10 wt %. (c) Schematic of sample preparation for multilayer hydrogel experiments. (d) Heatmap illustrating the spatial distribution of elastic modulus in heterogeneous hydrogels formed by combining different thiol–ene ratios and polymer concentrations (*n* = 3). (e) SEM images and (f) swollen hydrogels images after lyophilization, showing intact layered structures. (g) Layered hydrogel for constructing a 3D architecture; before and after immersion in PBS, showing integrity and adhesion between layers.

### Strength of Adhesion—(Peel Test)

To determine the adhesion properties between the layered hydrogels, the hydrogel was backed with tape, and the adhesion strength was measured using a T-peel test (Figure 3b). The bilayer hydrogels with polymer concentrations of 5, 7.5, and 10 wt % reached interfacial bonding forces of 0.02, 0.06, and 0.89N, respectively, before tearing. The failure mode observed in all bilayer hydrogel samples was complete tearing, rather than delamination at the interface.^44^ The fact that complete tearing occurred regardless of polymer concentration suggests that while adhesion was strong enough to prevent delamination, the hydrogel’s intrinsic mechanical strength remained the primary failure point. Specifically, in the T-peel test, the peel arm initially resisted separation, and as force increased, the hydrogel tore completely rather than peeling apart. This indicates that the adhesion between the hydrogel layers exceeded the material’s inherent tensile strength, leading to failure within the bulk of the hydrogel rather than at the interface.

### Stiffness Distribution and Adhesion Stability in Layered Gel

Nanoindentation experiments were performed to determine the Young’s modulus (stiffness) of multilayer thiol–ene hydrogels fabricated at 5, 7.5, and 10 wt % polymer content with thiol–ene ratios ranging from 0.6 to 1.6. The summarized heatmap (Figure 3d) illustrates a spatially controlled stiffness profile within the hydrogels. Layers with higher thiol content exhibited significantly lower Young’s modulus, with the thiol–ene ratio of 0.6 producing the highest modulus and the ratio of 1.6 consistently possessing the lowest modulus across all polymer concentrations. These findings align with the storage modulus (*G*′) trends observed in single-layer hydrogels with varying thiol–ene ratios (Figure 2b). For homogeneous linear elastic hydrogels, it is expected that the Young’s modulus E and shear modulus G are correlated according to E = 2G­(1 + ν), where ν is Poisson’s ratio = 0.5. Minor differences, however, may arise because rheological tests are performed under oscillatory shear, whereas nanoindentation was performed under uniaxial compression and conducted in liquid, where hydrogel swelling may contribute to reduced stiffness.^45^

The layered gels were frozen at −80 °C and subsequently lyophilized under vacuum. SEM images were then obtained using a JEOL scanning electron microscope. The lyophilized samples were further immersed again in PBS for 24 h. The results (Figure 3e,f) demonstrate that even after treatments such as freezing, lyophilization, and subsequent swelling, the hydrogel layers remained intact without detachment. Furthermore, different hydrogel architectures were fabricated and subsequently swollen in PBS for 48 h. The architecture remained intact, demonstrating the strong interlayer adhesion achieved with this method.

### In Vitro Cytotoxicity Studies

Evaluating cytotoxicity is the initial step in assessing the safety of biomaterials’ usage. To ensure that these PEG gels are well tolerated by mammalian cells, we conducted biocompatibility studies using the NIH/3T3 mouse embryonic fibroblast cells designed to evaluate acute cytotoxicity to the fabricated hydrogels following sterilization.

As shown in Figure 4, cell viability values were >90% for all formulations tested, with no significant difference observed between the different groups (*p* > 0.05). This outcome was expected, as PEG is widely recognized for its bioinertness, hydrophilicity, and resistance to protein adsorption.

**Figure 4:**
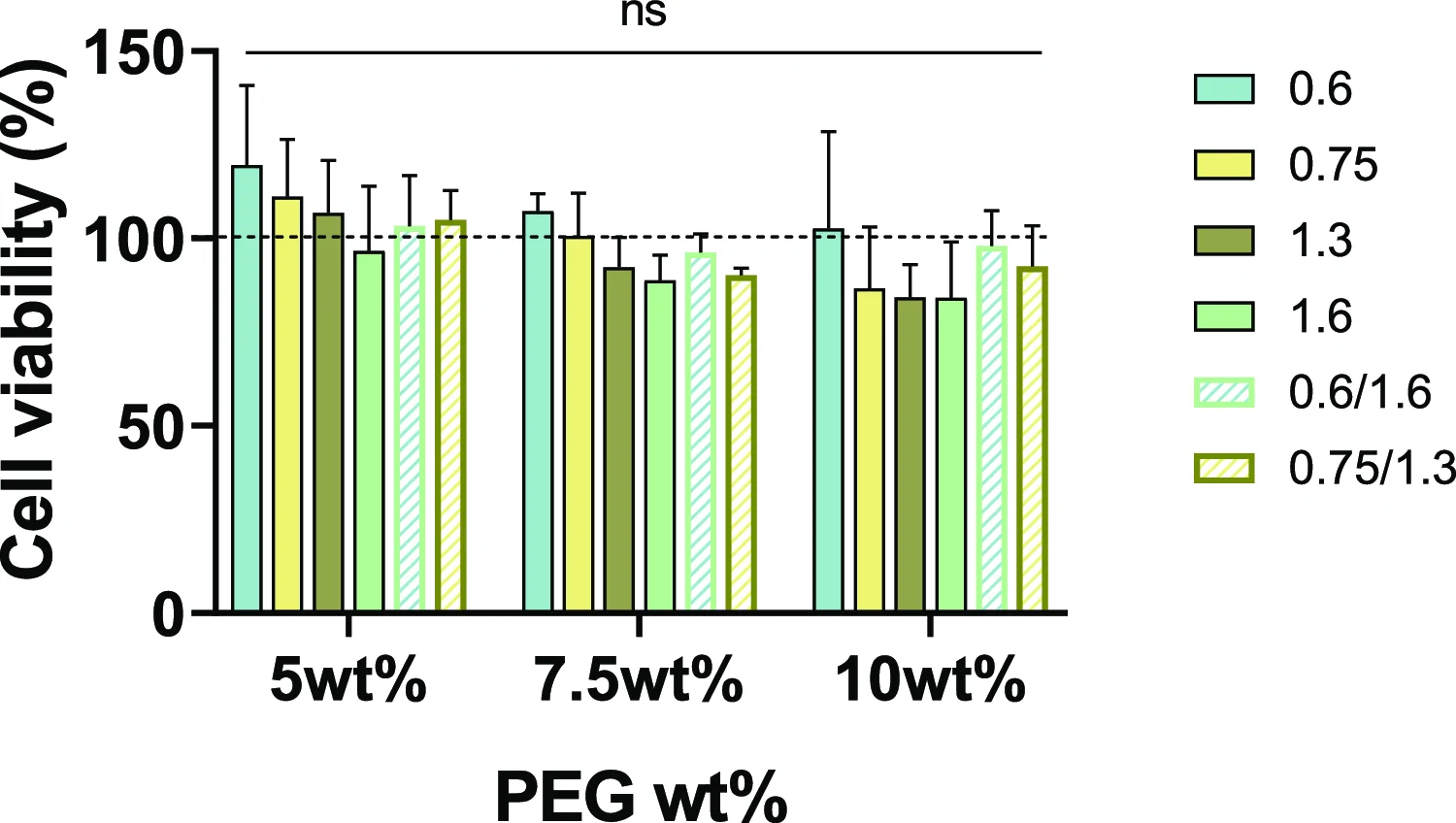
Cell viability of PEG hydrogels with varying PEG concentrations (5, 7.5, and 10 wt %) and thiol–ene ratio formulations (0.6, 1.3, 1.6, 0.6/1.6, and 0.75/1.3). All groups maintained high viability (>90%), with no statistically significant differences observed between conditions. Data are presented as mean ± standard deviation (*n* = 3).

The variation in PEG wt % represents differences in cross-linking density and mechanical stiffness of the hydrogels. Since higher PEG content increases the network density and stiffness, it could hinder nutrient diffusion or induce stress at the cell–material interface.^46^ However, the results suggest that even at the highest tested concentration (10 wt %), the hydrogel matrix did not create a cytotoxic environment. Similarly, varying the thiol–ene ratio modulates the cross-linking chemistry, where a balanced ratio (1:1) leads to an ideal network while deviations (e.g., 0.6 or 1.6) create thiol- or ene-rich environments. These off-stoichiometric ratios resulting in unreacted functional groups could potentially lead to altered mechanical/chemical microenvironments.^46^ Nonetheless, the lack of cytotoxic effects observed herein suggests that either the degree of residual reactivity was minimal or that any unreacted species was not present in cytotoxic concentrations. The bilayer hydrogel configurations (0.6/1.6 and 0.75/1.3) were also well tolerated by the cells. These constructs introduce an interface with different mechanical and chemical gradients, which might raise concerns regarding localized stress or leaching of reactive groups. However, the results indicate that such bilayer designs do not adversely affect cell viability after 24 h of exposure. Overall, the results indicate that these PEG hydrogels regardless of the polymeric concentrations, thiol–ene ratio, or layers, exhibited high cell viability and no evidence of acute cytotoxicity under the conditions tested.

These findings are limited to short-term cytocompatibility, and additional studies over longer culture periods will be necessary to evaluate long-term cellular responses. PEG-based networks are generally bioinert and do not inherently support cell attachments due to the absence of cell-adhesive motifs. Consequently, long-term cell adhesion, spreading, and proliferation would likely require incorporation of bioactive ligands such as RGD peptides or other extracellular matrix-derived cues.^47–49^

### Effects of Microparticles Embedded in the Hydrogel Matrix

Composite hydrogel-microparticle systems have been studied extensively for various biomedical applications such as drug delivery systems, tissue engineering, biosensing, and so on. These systems provide multifunctional advantages, including enhanced mechanical integrity, controlled release of bioactive agents, and improved stability.^50^ Among various microparticle types, alginate microparticles are particularly attractive due to their biocompatibility, biodegradability, and mild ionic cross-linking mechanisms.

Previous studies have successfully demonstrated the potential of alginate microparticles in various hydrogel platforms. For instance, Tring et al. encapsulated alginate microparticles encapsulated with bovine serum albumin (BSA) and transforming growth factor β3 (TGF_β3) into alginate microparticles using microfluidics and incorporated them into collagen scaffolds to promote sustained release during culturing human mesenchymal stem cells (MSCs) culture.^51^ Similarly, McShane et al. encapsulated porphyrin dyes and enzymes within alginate microparticles to develop biosensors for detecting glucose and oxygen by dispersing them in hydrogel matrices.^52–54^ Motivated by these applications, we incorporated alginate microparticles into our thiol–ene-based PEG hydrogels to evaluate their impact on physical properties such as swelling behavior and viscoelasticity.

Figure 5 presents the swelling ratios (left column) and storage moduli (right column) of PEG hydrogels with varying thiol–ene ratios (0.6, 0.75, 1.3, and 1.6), both with and without alginate microparticles. Incorporation of alginate microparticles resulted in a significant reduction in swelling ratio across all PEG concentrations. This effect was significantly pronounced at higher thiol–ene ratios. The observed reduction in swelling ratio upon microparticle incorporation is consistent with prior findings in composite hydrogel systems.^50,54^ Swelling behavior reflects the hydrogel’s network structure and cross-linking density. The presence of alginate microparticles likely acts as a physical filler within the matrix, effectively reducing the space available for water retention.

**Figure 5:**
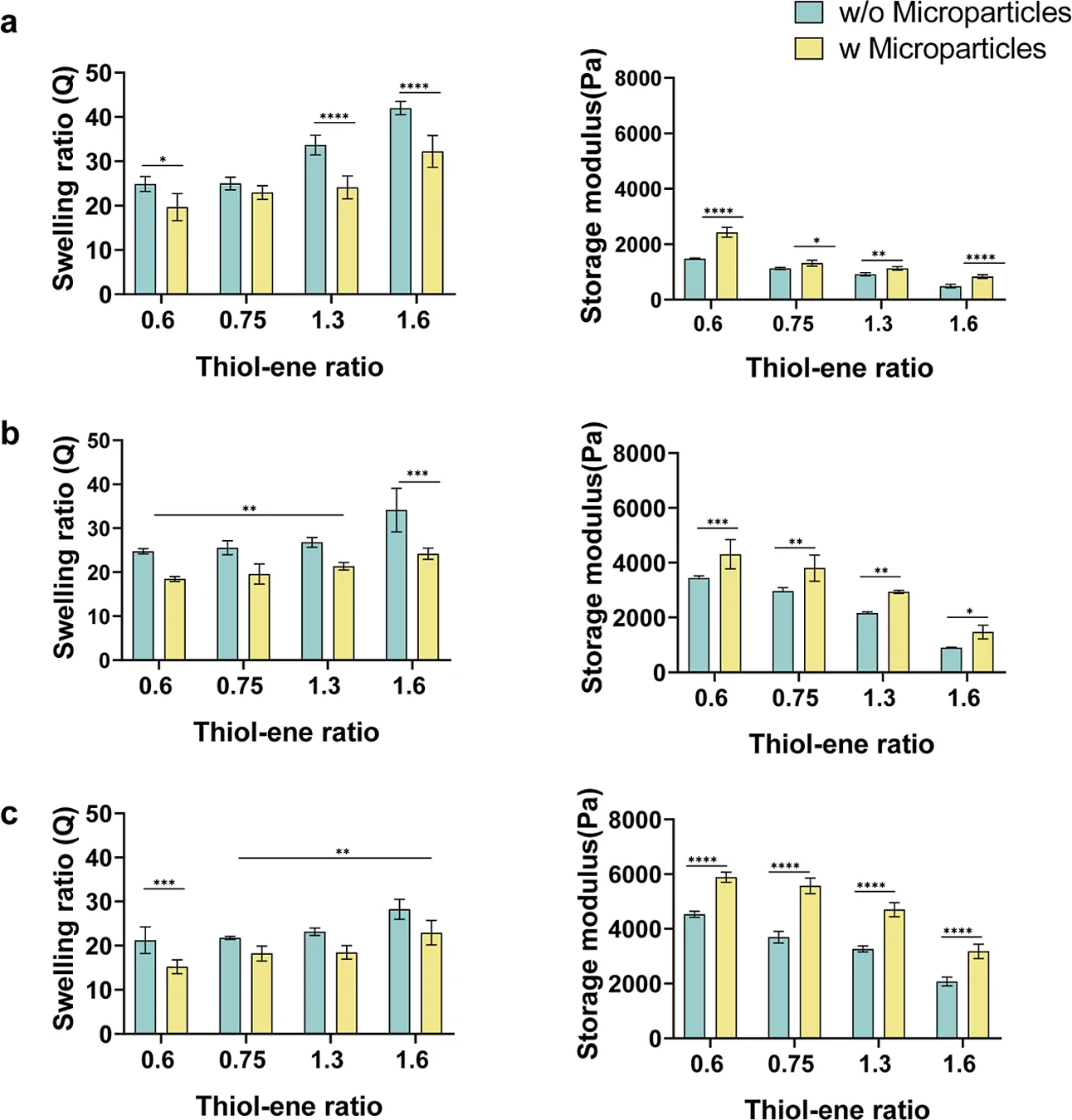
Swelling ratios (left) and storage moduli (right) of PEG-thiol–ene hydrogels formulated at different thiol–ene ratios (0.6, 0.75, 1.3, and 1.6) and polymer concentrations (a) 5, (b) 7.5, and (c) 10 wt %, with and without alginate microparticles. Error bars represent standard deviation (*n* = 4). Statistical significance is indicated: *p < 0.05; **p < 0.01; ***p < 0.001; ****p < 0.0001.

The storage modulus either increased or remained unchanged depending on the thiol–ene ratio and PEG content. For 7.5 and 10 wt % PEG formulations, incorporation of microparticles led to a marked increase in modulus, especially at higher thiol–ene ratios. This inverse relationship between swelling and stiffness indicates that microparticle addition promotes denser and more mechanically robust hydrogel networks. Together, these findings suggest that microparticles may be used strategically to tune the mechanics and water retention capacity of these thiol–ene hydrogels for biomedical applications.^50,55^

## Conclusion

A multilayer PEG hydrogel fabrication approach using off-stoichiometric thiol–ene chemistry was developed, resulting in independent adjacent layers that bonded without the need for UV light, photoinitiators, or external adhesives. These resulting hydrogels exhibited tunable gelation times, stiffness, swelling, and degradation while maintaining strong adhesion between layers. Mechanical testing confirmed that the interfacial bonding was stronger than the bulk material itself. Cytocompatibility studies showed high NIH/3T3 fibroblast viability across all formulations after 24 h of exposure. We also observed that inclusion of microparticles provided another lever to adjust the overall hydrogel swelling and stiffness. The layer-by-layer fabrication approach described herein enables the construction of composite hydrogel architectures with spatially defined material properties and preserved interfacial integrity. Such architectures are relevant to applications requiring spatial separation of functional components including multiplexed sensing and multiagent controlled release systems with independently tunable temporal profiles.
